# Sexually transmitted infections and associated risk factors among sexual minority women in China

**DOI:** 10.1038/s41598-023-48745-7

**Published:** 2023-12-07

**Authors:** Xiaofang Wang, Zhaohui Ouyang, Enwu Liu, Mengjie Han

**Affiliations:** 1 National Center for AIDS/STD Control and Prevention, Chinese Center for Disease Control and Prevention, 155 Changbai Road, Changping District, Beijing, 102206 China; 2 Jinsong Community Hospital, Chaoyang District, Beijing, China; 3 Mary Mackillop Institute for Health Research, Australian Catholic University, Melbourne, Australia; 4 College of Medicine and Public Health, Flinders University, Adelaide, Australia

**Keywords:** Diseases, Risk factors

## Abstract

There is a potential for transmission of sexually transmitted infections (STIs) within sexual minority women (SMW) in China. However, research specifically focused on STIs among SMW in China is severely limited. This study aims to evaluate the prevalence of STIs and identify associated risk factors among SMW in Beijing, China. This study comprised a baseline assessment followed by a follow-up evaluation. Consistent questionnaire interviews and STI tests were administered during both stages. Participants were recruited online in Beijing between 2020 and 2021 and factors associated with STIs were analyzed using logistic and Cox regression models. The baseline included 219 SMW, and 58.9% (129/219) of these individuals participated in the follow-up. During the baseline assessment, 4.1% (9/219) tested positive for chlamydia infection, while 5.0% (11/219) were HSV-2 seropositive. At the follow-up, the incidence of HSV-2 was 3.7 cases per 100 person-years. Notably, engaging in sexual activity with men and having an increased number of sexual partners were both identified as factors associated with a higher risk of STIs. The findings suggest that SMW in Beijing may face a significant risk of contracting STIs. As a preventive measure, there should be a concerted effort to promote STI testing within the SMW community.

## Introduction

Studies on the sexual health of sexual minority women (SMW), encompassing both women who have sex with women (WSW) and those who engage in sexual activity with both women and men (WSW/M), have been extensively documented in countries such as the US, UK, and Australia, yet they remain relatively scarce in the Chinese context^1–6^. Research has demonstrated the potential transmission of sexually transmitted infections (STIs), such as chlamydia, bacterial vaginosis, and HSV-2, within female-female sexual encounters^4,7–9^. When compared to sexual majority women, STI prevalence among the SMW population can vary, showing either an increase^9^ or a decrease^10^. Notably, within the SMW demographic, individuals identifying as WSW/M exhibit a higher susceptibility to STIs compared to exclusive WSW^6,8,9^.

China is believed to possess one of the largest populations of SMW^11^. While investigations into the mental health^12^, breast health^13^, and general healthcare^14^ of Chinese SMW have been reported, the principal investigator (PI) of this study stands as the sole researcher to have conducted a sexual health study encompassing sexual behaviour and STI prevalence among SMW in China during the years 2010–2011^15,16^. The earlier study revealed a high STI prevalence, albeit hampered by limited funding which impacted the accuracy of gonorrhea and chlamydia diagnostic methods. To obtain a precise evaluation of STI risk within the Chinese SMW population, the current study employed high-accuracy testing methodologies to gauge STI prevalence and incidence, along with an exploration of the associated risk factors.

## Materials and methods

This research comprised two stages: a baseline assessment and a subsequent follow-up. Subsequent to the baseline, all participants were invited to partake in the follow-up study, which took place approximately one year later. The baseline phase was conducted between September 2020 and July 2021, while the follow-up spanned from October 2021 to March 2022. The questionnaires and STI tests employed during both the baseline and follow-up stages remained consistent.

### Study site

The study was carried out at Jinsong Community Hospital, situated within the Chaoyang District of Beijing, China. The hospital's strategic location near the East Second Ring Road, in close proximity to the center of Beijing, facilitated convenient transportation.

### Sample size calculation

The required sample size was determined using the formula: $\mathrm{N}=\frac{{{U}^{2}}_{\mathrm{\alpha }/2}\pi \left(1-\pi \right)}{{\delta }^{2}}$.

Here, α = 0.05, U_α/2_ = 1.96, δ = 0.05 and the parameter π (prevalence) was set to 0.21. This prevalence value was sourced from a prior study on gonorrhea and chlamydia prevalence among WSW in Beijing during 2010–2012^16^. Based on these assumptions, the calculated baseline sample size required was 255 SMW.

### Eligibility criteria for the study

(1) Participants must be of biological female sex, (2) age of 18 years or older, (3) current residence in Beijing, and (4) engagement in sexual activity with another woman at least once. Sexual activity between women was defined to include genital contact such as oral, clitoral, vaginal, or anal intercourse. The term 'SMW' in this study was employed to encompass both WSW and women who have sex with women and men (WSW/M).

### Baseline

**Figure 1:**
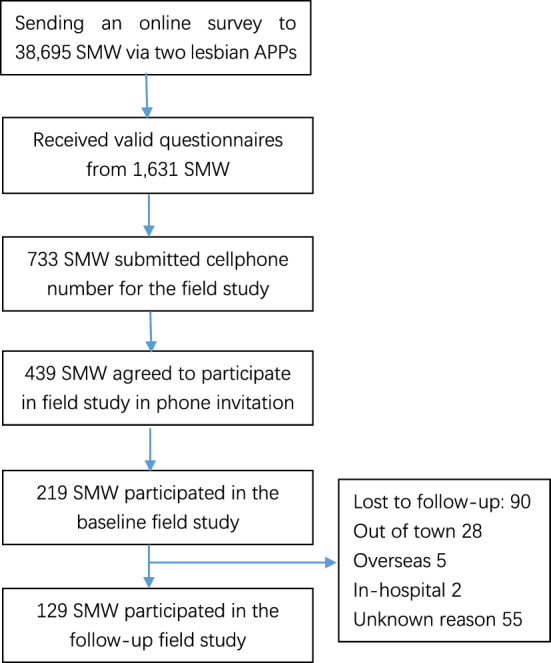
Flow chart of participant recruitment.

Participants for the baseline were recruited from a pool of 1631 SMW who had previously completed an online cross-sectional study facilitated by Rela and Lesdo, two of the most widely used smartphone apps for SMW in China in 2018 (see Fig. 1). These two apps encompassed a total of 38,695 registered SMW who had engaged in online activities within Beijing between June 1st, 2019 and May 31st, 2020. In the context of the online cross-sectional study, participants completed a questionnaire, and those expressing interest in undergoing an offline STI test were requested to provide their cellphone number. Subsequently, the study's principal investigator (PI) XW extended invitations for the offline test to each willing participant via phone. All SMW involved in the baseline phase were offered onsite STI tests; and their submitted online questionnaires were reviewed by the PI (XW).

### Follow-up

All SMW involved in the baseline phase were invited to take part in the follow-up questionnaire interview and STI tests. Follow-up questionnaires were conducted through face-to-face interviews with either the PI (XW) or another trained interviewer, conducted in private rooms. After completion, all questionnaires were reviewed by one of the two interviewers who had not conducted the initial interview with the respective SMW. Then they were offered onsite STI testing.

### Questionnaire

The questionnaire collected a range of information, including socio-demographic characteristics, STI risk factors such as sexual history (including specific sexual behaviors, number of sexual partners, G-spot stimulation, and heterosexual intercourse within the past year), history of STI testing, and willingness for childbearing.

The questionnaire employed for both the baseline and follow-up studies was based on instruments utilized in a previous study targeting WSW, conducted by the PI^15^. Following discussions with key informants and focus group discussions involving SMW, the questionnaire was modified. A preliminary version of the questionnaire was tested with 15 SMW, and the feedback gathered from this pilot phase was integrated into the finalized questionnaire.

### STI testing

STI testing encompassed both a blood test and a gynecological examination. A trained nurse collected 5 ml of whole blood from each participant for the blood test. The serum was then separated via centrifugation to diagnose herpes simplex virus type 2 (HSV-2) IgM and IgG (ELISA; Trinity, Inc, Ireland), HIV (ELISA; Jinhao, Inc, Beijing, PRC), and syphilis (Treponema pallidum particle agglutination test, Fujifilm Rebio, Japan; Toluidine red untreated serum test, Robio, Inc, Shanghai, PRC). Gynecological examinations followed the instructions provided by the test kits. Trained female Gynecologists collected vaginal secretions for trichomonas and candidiasis, with the secretions tested using the wet mount and gram stain methods. Cervical swabs were collected for chlamydia trachomatis and Neisseria gonorrhoeae testing, utilizing real-time PCR assay (Cobas 4800 system, Roche, Switzerland). All participants received post-test counselling, and those diagnosed with STIs received treatment at the study hospital or were referred to specialized hospitals or clinics.

### Data statistics and analysis

The baseline survey data were retrieved from the online database and then carefully reviewed in Microsoft Excel for accuracy. The follow-up survey data were subjected to a double-entry process using Epidata3.1 (The Epidata Association Odense, Odense, Denmark) to ensure precision. Statistical analyses were carried out using SPSS 17.0TM (SPSS Inc., Chicago, IL, USA).

Categorical variables are expressed as counts and percentages, and their analysis was conducted using Pearson's χ^2^ tests or Fisher's exact tests. Continuous variables are presented as medians and interquartile ranges (IQRs). Descriptive statistics were separately presented for both WSW and WSW/M groups. Initial univariable logistic regression was applied to all demographic and behavioral characteristics as independent variables. Variables exhibiting P-values ≤ 0.1 in the univariable analysis were subsequently incorporated into the multivariable analysis.

Unadjusted odds ratios (ORs) and adjusted odds ratios (AORs), along with their corresponding 95% confidence intervals (CIs), were calculated through both univariable and multivariable logistic regression models. The relationship between baseline characteristics and HSV-2 incidence was explored using Cox regression models. To assess potential significant differences in demographic characteristics between participants who attended the follow-up and those lost to follow-up, a χ^2^ test was performed.

All reported P-values are two-sided, and those ≤ 0.05 were deemed statistically significant.

### Ethics

Informed consent was procured from all participants. Each participant thoroughly read and signed an online informed consent document. Participation in the study was completely voluntary and anonymous, ensuring the privacy and autonomy of each participant.

The study received ethical approval from the institutional review board of the National Center for AIDS/STD Control and Prevention, and was conducted in accordance with their regulations. The approval number for this study is X191030591.

### Ethical approval statement

The study was approved by the institutional review board of the National Center for AIDS/STD Control and Prevention. The approval number is X191030591.

## Results

### Sociodemographic characteristics at baseline

The study sample was drawn from a pool of 1,631 sexual minority women (SMW) who had previously taken part in an online cross-sectional study facilitated by Rela and Lesdo. These two smartphone apps were among the most widely utilized platforms for SMW in China. Among the total pool of 1,631 SMW, the majority (97.2%, 1,585/1,631) were affiliated with Rela, while a smaller portion (2.8%, 46/1,631) were associated with Lesdo.

Out of the 1,631 SMW, approximately 44.9% (733/1,631) expressed willingness to participate in the field study and, as an initial step, shared their cellphone numbers through the online study platform. The principal investigator (PI) of the study then proceeded to reach out to all 733 of these SMW, inviting them to partake in the field study. Ultimately, 59.9% (439/733) of those invited accepted the invitation, and from this group, a total of 49.9% (219/439) ultimately participated in the study (as illustrated in Fig. 1). The demographic details, sexual behavior, testing behavior, and sexual health attributes of all SMW are presented in Table 1.

**Table 1:** Characteristics of sexual minority women at baseline in Beijing, China.

Characteristic	No. (%) of SMW (N = 219)	No. (%) of WSW/M in the past year^a^ (N = 34)	No. (%) of WSW exclusive in the past year^b^ (N = 185)	P value
Demographics				
Age (years)				
Median (IQR)	27 (24–32)	27 (24–29)	27 (24–32)	
20–29	142 (64.8)	26 (76.5)	116 (62.7)	0.313
30–39	63 (28.8)	7 (20.6)	56 (30.3)	0.663
40–57	14 (6.4)	1 (2.9)	13 (7.0)	
Ethnicity				
Han Chinese	198 (90.4)	32 (94.1)	166 (89.7)	0.543*
Other	21 (9.6)	2 (5.9)	19 (10.3)	
Beijing local residence				
Yes	77 (35.2)	11 (32.4)	66 (35.7)	0.709
No	142 (64.8)	23 (67.6)	119 (64.3)	
Education				
Junior high school or below	1 (0.5)	0 (-)	1 (0.5)	1.000
Senior high school	8 (3.7)	0 (-)	8 (4.3)	1.000
Undergraduate	155 (70.8)	26 (76.5)	129 (69.7)	0.700
Graduate or above	55 (25.1)	8 (23.5)	47 (25.4)	
Marital status				
Unmarried, living alone or with parents	141 (64.4)	25 (73.5)	116 (62.7)	0.499
Married (non-contract)	3 (1.4)	3 (8.8)	0 (0)	0.999
Married (contract)	3 (1.4)	0 (0)	3 (8.8)	0.999
Unmarried, living with a female sex partner	67 (30.6)	3 (8.8)	64 (34.6)	0.082
Unmarried, living with a male sex partner	2 (0.9)	2 (5.9)	0 (0)	0.999
Divorced, or widowed	3 (1.4)	1 (2.9)	2 (1.1)	
Employment status				
Student	68 (31.1)	14 (41.2)	54 (29.2)	0.693
Employed full-time	119 (54.3)	11 (32.4)	108 (58.4)	0.662
Freelance	25 (11.4)	8 (23.5)	17 (9.2)	0.372
Unemployed	7 (3.2)	1 (2.9)	6 (3.2)	
Monthly income				
No income	41 (18.7)	8 (23.5)	33 (17.8)	0.290
< CNY 5,000	44 (20.1)	11 (32.4)	33 (17.8)	0.092
CNY 5001–10,000	82 (37.4)	9 (26.5)	73 (39.5)	0.920
≥ CNY 10,000	52 (23.7)	6 (17.6)	46 (24.9)	
Self-reported sexual orientation				
Homosexual	152 (69.4)	9 (26.5)	143 (77.3)	0.465
Heterosexual	2 (0.9)	1 (2.9)	1 (0.5)	0.272
Bisexual	57 (26.0)	23 (67.6)	34 (18.4)	0.158
Other^c^	8 (2.7)	1 (2.9)	7 (3.8)	
Planned ways to give birth to a baby				
Already had a baby	3 (1.4)	0 (0)	3 (1.6)	0.999
Artificial insemination	49 (22.4)	8 (23.5)	41 (22.2)	0.457
Give birth in a wedlock	13 (5.9)	8 (23.5)	5 (2.7)	< 0.001
Surrogacy	6 (2.7)	0 (0)	6 (3.2)	0.999
No plan to give birth to a baby	148 (67.5)	18 (52.9)	130 (70.3)	
Sexual behavior				
Sex of the first sexual partner				
Female	166 (75.8)	16 (47.1)	150 (81.1)	< 0.001
Male	53 (24.2)	18 (52.9)	35 (18.9)	
Age of female-female sexual debut				
Median (IQR)	19 (18–22)	21 (19–23)	19 (18–22)	
13–15	14 (6.4)	1 (2.9)	13 (7.0)	0.296
16–22	159 (72.6)	24 (70.6)	135 (73.0)	0.469
23–37	46 (21.0)	9 (26.5)	37 (20.0)	
Number of sexual partners ever				
1	40 (18.3)	0 (0)	40 (21.6)	0.997
2–5	124 (56.6)	21 (61.8)	103 (55.7)	0.294
> 6	55 (25.1)	13 (38.2)	42 (22.7)	
Number of sexual partners in the past year				
0	16 (7.3)	0 (0)	16 (8.6)	0.998
1	143 (65.3)	8 (23.5)	135 (73.0)	< 0.001
2–5	53 (24.2)	20 (58.8)	33 (17.8)	0.040
> 6	7 (3.2)	6 (17.6)	1 (0.5)	
Specific sexual behaviors in the past year				
Oral sex	158 (72.1)	27 (79.4)	131 (70.8)	0.304
Vaginal sex	217 (99.1)	34 (100.0)	183 (98.9)	1.000
SM	24 (11.0)	9 (26.5)	15 (8.1)	0.004*
Group sex	2 (0.9)	1 (2.9)	1 (0.5)	0.287*
Anal sex	23 (10.5)	9 (26.5)	14 (7.6)	0.003*
Number of female sexual partners in the past year				
0	35 (16.0)	9 (26.5)	26 (14.1)	0.829
1	137 (62.6)	13 (38.2)	124 (67.0)	0.240
2–3	38 (17.4)	10 (29.4)	28 (15.1)	0.800
> 3	9 (4.1)	2 (5.9)	7 (3.8)	
Specific female-female sexual behaviors in the past year				
Digital-clitoral contact	165 (75.3)	24 (70.6)	141 (76.2)	0.484
Digital-vaginal contact	171 (78.1)	24 (70.6)	147 (79.5)	0.250
Oral-clitoral contact	141 (64.4)	23 (67.6)	118 (63.8)	0.665
Oral-vaginal contact	100 (45.7)	19 (55.9)	81 (43.8)	0.193
Clitoral-clitoral contact	53 (24.2)	10 (29.4)	43 (23.2)	0.440
Use sex toys with clitoris	86 (39.3)	13 (38.2)	73 (39.5)	0.893
Use sex toys with vagina	69 (31.5)	14 (41.2)	55 (29.7)	0.187
Used a sex toy with female sexual partners in the past year				
Yes	125 (57.1)	20 (58.8)	105 (56.8)	0.823
No	94 (42.9)	14 (41.2)	80 (43.2)	
Shared a sex toy with female sexual partners in the past year				
Yes	73 (33.3)	13 (38.2)	60 (32.4)	0.509
No	146 (66.7)	21 (61.8)	125 (67.6)	
Stimulating G-spot during female –female sex				
Yes	131 (59.8)	23 (67.6)	108 (58.4)	0.311
No	88 (40.2)	11 (32.4)	77 (41.6)	
Seeking female sex partners via internet				
Yes	132 (60.3)	23 (67.6)	109 (58.9)	0.339
No	87 (39.7)	11 (32.4)	76 (41.1)	
Ever taking alcohol during sex in the past year				
Yes	76 (34.7)	15 (44.1)	61 (33.0)	0.210
No	143 (65.3)	19 (55.9)	124 (67.0)	
Sexual health				
Main symptoms during female-female sex in the past year (N = 184)				
Increased leucorrhea	36 (19.6)	5 (20.0)	31 (19.5)	1.000*
Bellyache	16 (8.7)	2 (2.0)	14 (8.8)	1.000*
Vulvar discomfort	51 (27.7)	6 (24.0)	45 (28.3)	0.655
Bleeding	36 (19.6)	4 (16.0)	32 (20.1)	0.629
No symptoms	87 (47.3)	13 (52.0)	74 (46.5)	0.611
Ever had symptoms of painful urination, abnormal vaginal discharge, or genital ulcer in the past year				
Yes	59 (26.9)	14 (41.2)	45 (24.3)	0.042
No	160 (73.1)	20 (58.8)	140 (75.7)	
Ways to cope with the symptoms of painful urination, abnormal vaginal discharge, or genital ulcer (N = 59)				
See a doctor	23 (39.0)	5 (35.7)	18 (40.0)	0.774
Purchase non-prescription drugs	24 (40.7)	3 (21.4)	21 (46.7)	0.093
Douching	29 (49.2)	5 (35.7)	24 (53.3)	0.249
Do nothing	14 (23.7)	3 (21.4)	11 (24.4)	0.817
Infected STIs^d^				
Chlamydia	9 (4.1)	7 (20.6)	2 (1.1)	< 0.001*
Herpes simplex virus type 2 IgM + (HSV-2 IgM +)	3 (1.4)	3 (8.8)	0 (0)	0.003*
Herpes simplex virus type 2 IgG + (HSV-2 IgG +)	8 (3.7)	1 (2.9)	7 (3.8)	1.000*
Syphilis IgM + and IgG +	1 (0.5)	0 (0)	1 (0.5)	1.000*
Trichomonas	1 (0.5)	0 (0)	1 (0.5)	1.000*
Candidiasis	1 (0.5)	1 (2.9)	0 (0)	1.000*
Infected with any STIs	16 (7.3)	8 (23.5)	8 (4.3)	0.001*
Risk perception and health seeking behavior				
Perceived risk of getting infected with sexually transmitted diseases via female-female sex				
No idea	14 (6.4)	1 (2.9)	13 (7.0)	0.373
Low	103 (47.0)	20 (58.8)	83 (44.9)	0.779
Medium	82 (37.4)	10 (29.4)	72 (38.9)	0.811
High	20 (9.1)	3 (8.8)	17 (9.2)	
Had tested for STIs ever				
Yes	137 (62.6)	25 (73.5)	112 (60.5)	0.150
No	82 (37.4)	9 (26.5)	73 (39.5)	
Had tested for STIs in the past year				
Yes	128 (58.4)	24 (70.6)	104 (56.2)	0.118
No	91 (41.6)	10 (29.4)	81 (43.8)	
Routes of seeking knowledge of STIs				
Internet	207 (94.5)	33 (97.1)	174 (94.1)	0.697*
Doctors	52 (23.7)	10 (29.4)	42 (22.7)	0.398
Free publicity materials	59 (26.9)	7 (20.6)	52 (28.1)	0.364
On campus	58 (26.5)	10 (29.4)	48 (25.9)	0.674

*SMW* sexual minority women, *IQR* inter-quartile range, *CNY* Chinese Yuan, *SM* sadomasochism, *STIs* sexually transmitted infections, *IgM* immunoglobulin M; *IgG* immunoglobulin G.

*Fisher exact test.

^a^“WSW/M in the past year” refers to women who had had sex with both women and men in the past year.

^b^“WSW exclusive in the past year” refers to women who had only had sex with women in the past year.

^c^Other includes four pansexual women, two asexual women, one woman of uncertain sexuality.

^d^One woman infected with trichomonas and HSV-2 IgG + , one syphilis (IgM + and IgG +) and HSV-2 IgG + , two chlamydia and HSV-2 IgM + , two chlamydia and HSV-2 IgG + , one chlamydia and candidiasis. Four women only infected with chlamydia, one HSV-2 IgM + , and four HSV-2 IgG +.

Out of the 219 sexual SMW who participated, the majority (90.4%) identified as ethnic Han, and 35.2% were local residents of Beijing. The average age of the participants was 29.0 years (with a standard deviation of ± 5.0), ranging from 20 to 57 years. Regarding marital status, 95.9% of the SMW were unmarried, while a small proportion (1.4%) were in contract marriages. About 31.1% of the participants were students. In terms of monthly income, over half (61.1%) reported earning at least 5,000 RMB (approximately 740 USD).

A substantial majority of the SMW (95.9%) had achieved at least a college-level education. Most participants identified as homosexual (69.4%) and expressed no intention of having a baby (67.5%). It's worth noting that only one participant reported having ever used illicit drugs.

### Sexual behavior at baseline

Only 24.2% reported their first sexual partner as male, while 72.6% reported their female-female sexual debut occurring between the ages of 16 and 22. Approximately 60.3% of participants sought female sex partners through the internet, with 59.8% reporting the stimulation of the G-spot during female-female sexual encounters. Around 81.7% stated they had engaged in sexual activities with two or more partners at some point. In the past year, 84.0% of the surveyed SMW reported engaging in sexual activity with another woman, and 21.5% had two or more female sex partners. The most commonly reported female-female sexual behaviors were digital-clitoral contact (75.3%) and digital-vaginal contact (78.1%). Conversely, the least popular behaviors were clitoral-clitoral contact (24.2%) and the use of sex toys involving the vagina (31.5%). Furthermore, 33.3% of participants indicated that they shared a sex toy with their female sex partners. About 27.4% reported having two or more sex partners in the past year. Specific sexual behaviors in the past year included oral sex (72.1%), vaginal sex (99.1%), sadomasochism (SM, 11.0%), group sex (0.9%), and anal sex (10.5%). Notably, 15.5% (34 out of 219) reported engaging in sexual activity with a man in the past year, and among these women, 58.8% (20 out of 34) consistently used condoms with their male partners.

### Sexual health in the past year at baseline

About 7.3% (16 out of 219) of the participants were found to be infected with various STIs, including HSV-2 (5.1%), candidiasis (0.5%), trichomonas (0.5%), chlamydia (4.1%), and syphilis (0.5%) (as indicated in Table 1). No cases of HIV or gonorrhea infection were identified among the respondents. Approximately one-third (37.4%) had never undergone STI testing, while 41.6% had not been tested for STIs in the past year. Roughly half (46.5%) of the SMW expressed a moderate to high perceived risk of contracting STIs through female-female sexual activities.

Among the 59 individuals (26.9%) who reported experiencing symptoms such as painful urination, abnormal vaginal discharge, or genital ulcers in the past year, 49.2% (29 out of 59) used vaginal douching (cleaning the vagina with water or gynecological lotion) as a response, and 23.7% (14 out of 59) took no action in response. In the context of female-female sexual encounters within the past year, 52.7% reported having experienced symptoms like bleeding, abdominal discomfort, increased vaginal discharge, or vulvar discomfort.

Table 1 also presents the characteristics of WSW (Women who have Sex with Women) and WSW/M (Women who have Sex with Women and Men) in the past year. In comparison to WSW, a higher proportion of WSW/M individuals self-identified as bisexual, had their first sexual experience with a man, engaged in sexual activities with multiple partners in the past year, participated in sadomasochism and anal sex, contracted STIs, and reported symptoms of painful urination, abnormal vaginal discharge, or genital ulcers in the past year. Conversely, more WSW individuals self-identified as homosexual, were unmarried, and were cohabiting with a female sex partner.

### Follow up

Out of the initial 219 SMW included at baseline, 58.9% (129 out of 219) took part in the follow-up study. Among the 129 SMW who participated in the follow-up, 9 (7.0%) tested positive for various STIs. Specifically, three new cases of HSV-2 (IgM +) infection were identified. Additionally, three individuals had HSV-2 (IgG +) infections, which were consistent with their baseline status. One person initially infected with HSV-2 (IgM +) was subsequently found to have both HSV-2 (IgM +) and HSV-2 (IgG +) infections during the follow-up period. Another person, who was infected with chlamydia at follow-up, was among the nine SMW who had chlamydia at baseline. Notably, she did not seek treatment for the infection between baseline and follow-up. One individual was newly diagnosed with candidiasis.

During the follow-up period, the 129 SMW were collectively observed for a total of 108 person-years. The infection rate for HSV-2 (IgM +) was calculated to be 3.7 cases per 100 person-years (4 cases out of 108 person-years).

Among the 90 SMW who were lost to follow-up, 28 were out of town, 5 were abroad, 2 were hospitalized, and the reasons for the remaining 55 being lost to follow-up were unknown. Baseline socio-demographic and behavioral characteristics were compared between the 129 SMW who participated in the follow-up and the 90 SMW who were lost to follow-up. The only notable difference was observed in the age at which they had their first female-female sexual experience (P = 0.012). On average, the age of SMW who attended the follow-up was 19.7 years (with a standard deviation of ± 3.5; ranging from 14 to 37 years), while the age of those who were lost to follow-up was 20.6 years (with a standard deviation of ± 3.4; ranging from 13 to 30 years).

### Factors associated with chlamydia

No new cases of chlamydia were identified during the follow-up period. Factors associated with chlamydia were investigated through logistic regression analysis at baseline, and the results are presented in Table 2.

**Table 2:** Factors associated with chlamydia among sexual minority women in Beijing.

Factor	n/N (%)	Unadjusted OR (CI)^a^	P-value^b^	AdjustedOR (CI)^a^	P-value^b^
Sex of the first sexual partner					
Female	4/166 (2.4)	0.2 (0.1–0.7)	0.007	0.4 (0.1–1.1)	0.075
Male	5/53 (9.4)	1.0		1.0	
Number of sexual partners in the past year					
0	0 (0)	0.1 (0.01–1.1)	0.060	0.4 (0.02–6.5)	0.485
1	1/143 (0.7)	0.1 (0.01–0.3)	0.001	0.2 (0.02–1.3)	0.091
2–5	6/53 (11.3)	0.2 (0.04–1.1)	0.065	0.3 (0.05–2.1)	0.240
> 6	2/7 (28.6)	1.0		1.0	
Had sex with a male in the past year					
Yes	7/34 (20.6)	6.0 (2.1–16.9)	0.001	6.0 (2.1–16.9)	0.001
No	2/185 (1.1)	1.0		1.0	

*OR* odds ratio, *CI* 95% confidence interval.

^a^Univariable logistic regression was performed with all characteristics as independent variables. Only variables with p-values < 0.1 in univariable analysis were included in the multivariable analysis.

^b^All p-values are 2-sided. Comparisons with p-values < 0.05 were considered statistically significant.

In the univariable logistic regression model, several significant independent factors for chlamydia were identified. These factors included having a female as the first sexual partner (Odds Ratio [OR] 0.2; 95% Confidence Interval [CI] 0.1–0.7), having only one sexual partner in the past year (OR 0.1; 95% CI 0.01–0.3), and engaging in sexual activities with a male partner in the past year (OR 6.0; 95% CI 2.1–16.9).

Upon further analysis using the multivariable model, it was found that the only significant independent factor associated with chlamydia was engaging in sexual activities with a male partner in the past year (Adjusted Odds Ratio [AOR] 6.0; 95% CI 2.1–16.9).

### The risk factor for HSV-2

During the follow-up period, three new cases of HSV-2 were identified. The risk factors associated with HSV-2 infection were analyzed using a Cox regression model, and the findings are detailed in Table 3.

**Table 3:** The risk factor for HSV-2 among sexual minority women in Beijing.

Factor	n/N (%)	Unadjusted HR (CI)^a^	P-value^b^	Adjusted HR (CI)^a^	P-value^b^
Had sex with a male in the past year					
Yes	3/15 (20.0)	21.66 (2.24–209.07)	0.008	21.7 (2.2–209.1)	0.008
No	1/114 (0.9)	1.0		1.0	

*HR* hazard ratio, *CI* 95% confidence interval.

^a^Univariable cox regression was performed with all characteristics as independent variables. Only variables with p-values < 0.1 in univariable analysis were included in the multivariable analysis.

^b^All p-values are 2-sided. Comparisons with p-values < 0.05 were considered statistically significant.

In both the univariable and multivariable Cox regression models, the sole significant risk factor was engaging in sexual activities with males (Hazard Ratio [HR] 21.7; 95% Confidence Interval [CI] 2.2–209.1).

### Factors associated with any STIs

The factors associated with any STIs were outlined in Table 4. In the univariable logistic regression model, several significant independent factors for STIs were identified. These factors included having a female as the first sexual partner (Odds Ratio [OR] 0.2; 95% Confidence Interval [CI] 0.1–0.6), having only one sexual partner in the past year (OR 0.06; 95% CI 0.01–0.3), and engaging in sexual activities with a male partner in the past year (OR 6.8; 95% CI 2.4–19.7).

**Table 4:** Factors associated with sexually transmitted infections among sexual minority women in Beijing.

Factor	n/N (%)	Unadjusted OR (CI)^a^	P-value^b^	Adjusted OR (CI)^a^	P-value^b^
Sex of the first sexual partner					
Female	7/166 (4.8)	0.2 (0.1–0.6)	0.004	0.3 (0.1–1.1)	0.053
Male	9/53 (17.0)	1.0		1.0	
Number of sexual partners in the past year					
0	0/16 (0)	–	0.998	–	0.998
1	6/143 (4.2)	0.06 (0.01–0.3)	0.001	0.2 (0.02–1.4)	0.093
2–5	7/53 (13.2)	0.2 (0.04–1.1)	0.065	0.3 (0.05–2.1)	0.241
> 6	3/7 (42.9)	1.0		1.0	
Had sex with a male in the past year					
Yes	8/34 (23.5)	6.8 (2.4–19.7)	0.001	4.8 (1.5–14.8)	0.007
No	8/185 (4.3)	1.0		1.0	
Ever had symptoms of painful urination, abnormal vaginal discharge, or genital ulcer in the past year					
Yes	8/59 (13.6)	2.6 (1.0–7.2)	0.059	2.6 (0.8–8.1)	0.111
No	8/160 (5.0)	1.0		1.0	

*OR* odds ratio, *CI* 95% confidence interval.

^a^Univariable logistic regression was performed with all characteristics as independent variables. Only variables with p-values < 0.1 in univariable analysis were included in the multivariable analysis.

^b^All p-values are 2-sided. Comparisons with p-values < 0.05 were considered statistically significant.

Further analysis using the multivariable logistic model, the only significant independent factor associated with any STIs was engaging in sexual activities with a male partner in the past year (Adjusted Odds Ratio [AOR] 4.8; 95% CI 1.5–14.8).

## Discussion

This study aimed to assess STI prevalence and incidence among Sexual Minority Women (SMW) in China. The study's findings indicated that the prevalence of STIs observed was lower than what was reported in previous studies conducted in 2010–2011^15,16^. No cases of HIV were identified, and only one person tested positive for syphilis. Similar trends of low HIV and syphilis infections were also observed in comparable studies conducted in the US and the UK^8^. Improved test accuracy for gonorrhea and chlamydia in this study, as compared to the 2010–2011 study, made direct comparisons challenging. Unfortunately, the study lacked previous data on HSV-2 incidence for comparison.

Comparisons with national and international studies revealed intriguing insights. A US national study reported a higher chlamydia infection rate (7.1%) among SMW aged 15–24 compared to women exclusively engaging in heterosexual activities (5.3%)^17^. A study conducted in Southwest China from 2019 to 2021 reported a chlamydia infection rate of 6.5% among 4,526 women attending a gynecological diagnosis and treatment center^18^. Certain studies in China documented higher chlamydia prevalence (around 10%) among women attending STI clinics before the pandemic. Consequently, the chlamydia prevalence (4.1%) among SMW was more aligned with the general female population in China. Conversely, the HSV-2 infection rate observed in the study was higher than a province-specific study conducted among general women aged 18–49 near Beijing^19^. An American study involving 392 WSW noted an association between older age and HSV-2 infection^20^, which contrasts with our study where the SMW population was relatively younger (average age of 29 years) and exhibited a lower infection rate.

Primary risk factors for chlamydia and/or HSV-2 infection among SMW were identified as engaging in sexual activities with males and having an increased number of sexual partners. Previous research has also highlighted sex with males as a risk factor for chlamydia infection among SMW^4^. The transmission dynamics of HSV-2 suggest that more sexual partners, both male and female, may increase the risk of transmission^20^. The prevalent sexual practice of digital-genital contact, which may involve "rough sex," could potentially lead to physical trauma and increased STI transmission among SMW and their male or female partners.

The study's findings were influenced by the strict anti-pandemic measures in Beijing, leading to a premature end of the follow-up period. Consequently, some SMW were lost to follow-up, and the pandemic-related measures likely impacted sexual activities and STI incidence. Prior research has indicated reduced sexual activity frequency during the pandemic^21^. Studies have shown that gonorrhea infections decreased during the pandemic^22^, and chlamydia prevalence significantly decreased in the US compared to 2019^23^. However, these observed decreases might also be due to a reduction in screening tests^24^. It is plausible that the pandemic and associated restrictions led to decreased STI transmission, particularly due to reduced multiple sexual partnerships.

The US Center for Disease Control and Prevention guidelines recommend annual STI screening, including HSV-2, gonorrhea, and chlamydia, for sexually active females under 25 years old^25^. However, only about half of the SMW in this study underwent STI testing in the past year, and no participants reported STI-related symptoms during the study. Given the heightened risk of asymptomatic STIs among sexually active SMW, annual STI testing is recommended in China. It is crucial for guidelines to recognize the diversity of sexual behaviours and orientations to ensure adequate testing coverage.

Study limitations encompass potential bias in participant recruitment through internet and smartphone apps. The sample selected may primarily represent individuals who are more active online, younger, and better educated. Information bias may arise during face-to-face questionnaire interviews, and social desirability could be a factor due to the sensitive nature of questions related to sexual behaviors and drug use. Additionally, the study's sample size was limited, resulting in wide confidence intervals for the estimated associations. Given these limitations, while the study provides valuable insights into STI prevalence and risk factors among SMW in China, caution should be exercised when generalizing the results to the wider population. Further research with larger and more diverse samples, along with comprehensive data collection methods, would help to refine and validate the findings.

In conclusion, this study indicates that SMW might face a higher risk of certain STIs, such as chlamydia or HSV-2 infection, compared to the general female population in China. Interventions to promote consistent condom use during sexual activities with men and annual STI testing are crucial for SMW. Additionally, STI testing guidelines should be inclusive and encompass the diverse spectrum of sexual behaviors and orientations among women.

## Data Availability

The datasets analysed during the current study are not publicly available but are available from the corresponding author on reasonable request.
